# Supplementary material for: SyNDI: synchronous network data integration framework
Source: BMC Bioinformatics. 2018 Nov 6;19:403. doi: 10.1186/s12859-018-2426-5 (PMC6219086; doi:10.1186/s12859-018-2426-5)
Supplement: Supplementary file 5 — Files of the example presented in Additional file 4. This additional file is a zip package that contains all files used in Additional file 4. Detailed descriptions of each of these files are presented in a README file included in this zip package. (GZ 30411 kb) [file 12859_2018_2426_MOESM5_ESM.gz › SyNDITools/Meme2Fimo/meme_4.9.0_4.tar.gz!/meme_4.9.0/website/html/help_format.html]

MEME Suite - Input formats


|  |  |
| --- | --- |
|  | MEME Suite The MEME Suite web application requires the use of JavaScript   Javascript doesn't seem to be available on your browser.  The preferred sequence format for MEME Suite programs is Pearson/Fasta (FASTA) format. For example,  `>ICYA_MANSE INSECTICYANIN A FORM (BLUE BILIPROTEIN)  GDIFYPGYCPDVKPVNDFDLSAFAGAWHEIAK  LPLENENQGKCTIAEYKYDGKKASVYNSFVSNGVKEYMEGDLEIAPDA  >LACB_BOVIN BETA-LACTOGLOBULIN PRECURSOR (BETA-LG)  MKCLLLALALTCGAQALIVTQTMKGLDI  QKVAGTWYSLAMAASDISLLDAQSAPLRVYVEELKPTPEGDLEILLQKW`  Sequences start with a header line followed by sequence lines. A header line has the character ``>'' in position one, followed by an unique name without any spaces, followed by (optional) descriptive text. After the header line come the actual sequence lines. Spaces and blank lines are ignored. Sequences may be in capital or lowercase or both.  The first word in the header line of each sequence, truncated to 24 characters if necessary, is taken as the name of the sequence. This name must be unique. Sequences with duplicate names will be ignored. (The first word in the title line is everything following the ">" up to the first blank.) The web versions of MEME Suite programs also accepts protein and DNA sequences in any of the following formats by converting them to Pearson/Fasta format. When using these formats, it is not possible to specify sequence weights.   - **Sequence formats that allow one or   more sequences:** - IG/Stanford, used by Intelligenetics and others - GenBank/GB, genbank flatfile format - NBRF format - EMBL, EMBL flatfile format - DNAStrider, for common Mac program - Fitch format, limited use - Pearson/Fasta, a common format used by Fasta programs and   others - Zuker format, limited use - Olsen, format printed by Olsen VMS sequence editor - Phylip3.2, sequential format for Phylip programs - Phylip, interleaved format for Phylip programs (v3.3,   v3.4) - MSF multi sequence format used by GCG software - PAUP's multiple sequence (NEXUS) format - PIR/CODATA format used by PIR - ASN.1 format used by NCBI **Sequence formats that only allow one sequence. These formats   cannot be used to input multiple sequences.** - GCG, single sequence format of GCG software (use MSF format   instead) - Plain/Raw, sequence data only (no name, document,   numbering)  **For MEME only** Sequence weights may be specified in the dataset file by special header lines where the unique name is "WEIGHTS" (all caps) and the discriptive text is a list of sequence weights. Sequence weights are numbers in the range 0 < w <=1. All weights are assigned in order to the sequences in the file. If there are more sequences than weights, the remainder are given weight one. Weights must be greater than zero and less than or equal to one. Weights may be specified by more than one "WEIGHT" entry which may appear anywhere in the file, but you must not put weights on lines that don't start with ">WEIGHT". When weights are used, sequences will contribute to motifs in proportion to their weights. Here is an example for a file of three sequences where the first two sequences are very similar and it is desired to down-weight them:  `>WEIGHTS 0.5 .5  >WEIGHTS 1.0  >seq1  GDIFYPGYCPDVKPVNDFDLSAFAGAWHEIAK  >seq2  GDMFCPGYCPDVKPVGDFDLSAFAGAWHELAK  >seq3  QKVAGTWYSLAMAASDISLLDAQSAPLRVYVEELKPTPEGDLEILLQKW`  The  ReadSeq program is used for converting sequences to FASTA format. ReadSeq is copyright 1990 by D. G. Gilbert, Biology Dept., Indiana University. |
